# The Validity of Bioelectrical Impedance Analysis Compared to a Four-Compartment Model in Healthy Adults: A Systematic Review

**DOI:** 10.3390/jfmk11010065

**Published:** 2026-01-31

**Authors:** Christopher J. Oliver, Luke Del Vecchio, Michelle Minehan, Mike Climstein, Nedeljka Rosic, Stephen Myers, Grant Tinsley

**Affiliations:** 1 Faculty of Health, Southern Cross University, Lismore, NSW 2480, Australia; christopher.oliver@scu.edu.au (C.J.O.); 2 Faculty of Health, Southern Cross University, Bilinga, QLD 4225, Australia; michael.climstein@scu.edu.au (M.C.); nedeljka.rosic@scu.edu.au (N.R.); 3 Research Institute for Sport & Exercise, The University of Canberra, Bruce, ACT 2617, Australia; 4 NatMed-Research, Evans Head, NSW 2473, Australia; 5 Department of Kinesiology & Sport Management, Texas Tech University, Lubbock, TX 79409, USA; grant.tinsley@ttu.edu

**Keywords:** body composition, equivalence, Bland–Altman

## Abstract

**Background**: The four-compartment (4C) model is a criterion method for evaluating body composition tools like bioelectrical impedance analysis (BIA). This systematic review assessed the clinical equivalence of BIA devices compared to the 4C model and explored limitations in using the 4C model as a criterion method. **Methods**: Twelve cross-sectional and baseline longitudinal studies involving healthy, weight-stable, non-athlete, non-pregnant adults were included. The primary outcome was a Bland–Altman analysis, with bias, limits of agreement, and proportional bias extracted from each paper. The study quality was evaluated using the AXIS tool. Due to the high variability across studies, a meta-analysis was not performed. **Results**: BIA devices generally performed poorly against the 4C model estimates of percentage body fat and fat-free mass. Across the 12 studies, mean bias for percentage body fat between BIA and the 4C model ranged from −3.5% to +4.4%, with limits of agreement typically spanning 15 to 20 percentage points. For fat-free mass, mean bias ranged from −3.9 kg to +1.8 kg, with limits of agreement often exceeding ±6 kg. These wide limits indicate non-equivalence at the individual level despite small mean differences. Differences in both BIA device design and variations in 4C methodology across studies may have contributed to these discrepancies. **Conclusions**: BIA estimates of percentage body fat and fat-free mass were overall not equivalent to the 4C model. Alternative criterion methods, such as MRI, and use of raw BIA data are recommended. Standardization of BIA devices is also needed for improved clinical and research use.

## 1. Introduction

### Body Composition Models

Body composition assessment is preferable to body mass index (BMI) in providing an estimation of body composition for more robust health and disease risk estimates. There is a range of body composition assessment techniques, including anthropometry, chemical analysis of tissue by chemical or in vivo neutron activation analysis, electrical impedance such as bioelectrical impedance analysis (BIA) or bioimpedance spectroscopy (BIS), ultrasound, and radiation imaging, computed tomography (CT), dual x-ray absorptiometry (DXA), and non-radiation imaging, i.e., magnetic resonance imaging (MRI) [1]. Many body composition techniques considered to be of high precision, e.g., MRI or CT, currently have limited clinical applicability, owing to their availability, cost, complexity in use, or radiation exposure [1].

Bioelectrical impedance analysis (BIA) is widely used to estimate body composition in commercial, clinical, and research settings, as it is non-invasive, cost-effective, safe, and does not require specialized training. There are many iterations of BIA machines, for example, single/multifrequency, supine/vertical, hand-to-hand, leg-to-leg, arm-to-leg, and whole-body/segmental configurations [2]. Commonly used body composition outputs from BIA include percentage body fat (%BF), fat mass (FM), and fat-free mass (FFM). These outputs are indirect estimates validated against other reference techniques, the most frequent being DXA, and the four-compartment (4C) model. However, while DXA is often used as a reference method to assess BIA devices, the 4C model is regarded as a criterion method [3].

**Figure 1:**
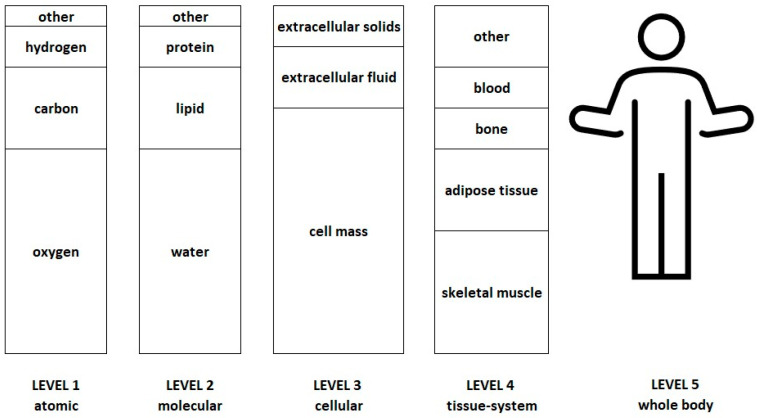
Five-level model of body composition, adapted from Wang et al. 1992 [6], not to scale.

The 4C model is a level 2 molecular model based on four distinct chemical components within the body, i.e., fat, water, minerals, and protein (Figure 1). Multiple 4C model equations have been derived by researchers using first principles, incorporating various assumptions, for example, the densities of tissues and the ratio of bone to non-bone masses [4]. The 4C model base equation expands on the 2C model of body mass (BM) = fat mass (FM) + fat-free mass (FFM) by providing better estimates of the FFM component, i.e., BM = FM + total body water (TBW) + bone mineral content (BMC) + residual. The base equation is rearranged so that the equations solve for FM and %BF. The popular 4C model equation by Wang et al. [5] is given as an example:

FM (kg) = 2.748 × (BV) − 0.699 × (TBW) + 1.129 × (BMC) − 2.051 × (BM)

%BF = (BM/FM) × 100

FFM = BM − FM

BM = Body Mass, BV = Body Volume, TBW = Total Body Water, Mo = bone mineral content.

Each of the components in the 4C model—total body water, body volume, and bone mineral content—requires measurement by criterion methods. However, the methods used to measure these components can vary between studies. For example, body volume could be estimated by either hydrodensitometry (underwater weighing), plethysmography (i.e., Bod Pod), or, more recently, in ‘rapid’ 4C models, by the use of DXA-derived volume estimates [7,8]. Bone mineral density is estimated by DXA, for which there are different makes, models, and software versions. Total body water can be measured by isotope dilution or bioimpedance spectroscopy (BIS). This means there are at least twelve possible permutations (3 body volume × 2 total body water × 1/2 bone mineral content) of the 4C model methods per equation. Yet, there is a paucity of research comparing estimates of body composition from BIA machines to 4C criterion models, particularly in examining the effects of different 4C model equations and their component methodology.

This review investigated studies in which percentage fat mass and fat-free mass estimates from bioelectrical impedance analysis (BIA) machines were compared to those from a criterion four-compartment (4C) model. The primary aim was to evaluate the level of agreement observed between BIA devices and the 4C model using the Bland–Altman limits of agreement analysis for percentage fat mass and fat-free mass. This review addresses the gap in the literature regarding the variability in 4C model equations and techniques, as well as the different configurations of BIA machines.

## 2. Methods

The study was registered in PROSPERO (CRD42023266802).

One of the authors searched PubMed, Medline (via EBSCO), SCOPUS, and CINAHL databases from inception until 31 May 2025, limiting the search criteria to English language, healthy adults, and papers that included cross-sectional data. The search string was designed for PubMed and translated for use in other databases using the Polyglot Search Translator [9]. The complete search strings for all databases are in Supplementary Table S1.

### 2.1. Study Selection and Screening

Two review authors independently screened the titles and abstracts for inclusion against the inclusion criteria; one author retrieved full texts, and the two authors screened the full texts for inclusion. Any disagreements were resolved by discussion or referred to a third author. Data extraction was conducted using standardized forms, and statistical summaries and calculations (including Bland–Altman metrics) were cross-checked for accuracy.

### 2.2. Inclusion Criteria

Design: Data was included from cross-sectional studies and baseline data from longitudinal studies.

Participants: Adults ≥ 18 years of age, healthy, non-pregnant women, non-athletes, and mass stable.

Outcome: The primary outcome was the percentage of body fat or fat-free mass from a 4C model compared to the same measure from bioelectrical impedance analysis. Studies must have used BIA machines as the comparator, and the 4-compartment models must have used DXA to estimate bone mineral content but not used DXA to obtain body volume. Studies must have used Bland–Altman limits of agreement (LOAs) in their accuracy analysis. When studies did not report LOAs, LOAs were calculated from the reported mean difference and standard deviation or estimated, if possible, from published Bland–Altman plots. Some studies had insufficient data to compute the bias and standard deviation; attempts were made to obtain this data if the papers were published within a reasonable timeframe.

Date: No limit publication date.

Language: English only.

### 2.3. Exclusion Criteria

Studies on children, pregnant women, athletes, or studies in which total body water in the 4C model was measured by a BIA machine were excluded. Studies or data from studies that compared changes in 4C model outcomes to BIA after intervention were excluded unless they had a baseline comparison. Papers that used Bioelectrical Impedance Spectroscopy (BIS) machines as a comparator to the 4C model were excluded, given the differences between BIA and BIS devices. However, papers that used BIS devices for the estimation of total body water as part of the 4C model were included.

### 2.4. Data Extraction

Data extraction included *participant demographics* (age, sex, race, and health status), data fields relating to the *BIA machine* (make, model, frequency type, and configuration, information relating to the validation of equations), *4C criterion method* (4C model used, techniques used for body volume, total body water, and bone mineral content) and *statistical methods* Bland–Altman Limits of Agreement (LOA). Data on proportional bias were extracted from LOA plots where these were available or from text.

### 2.5. Quality and Risk of Bias Assessment

Study quality was assessed independently by two authors using the Appraisal tool for Cross-Sectional Studies (AXIS), which is a series of twenty questions with a three-option response choice of ‘yes’, ‘no’, or ‘don’t know/comment’ [10]. Seven (Q1, 4, 10, 11, 12, 16, and 18) of the questions related to reporting quality; seven (Q 2, 3, 5, 8, 17, 19, and 20) of the questions related to study design quality; and six related to the possible introduction of biases in the study (6, 7, 9, 13, 14, and 15). Several questions relating to response rate (Q7, 13, 14) were excluded, as they were not relevant. The AXIS questionnaire does not provide an overall aggregate score. Given the importance of fluid and alcohol intake and strenuous exercise on hydration status, premeasurement restrictions on these factors were extracted from each study.

### 2.6. Primary Outcome Analysis

The primary endpoint was the individual comparability between the 4C model and the BIA machine for %BF or FFM (kg). The Bland–Altman approach, which plots the difference between methods against the mean of the two methods, was chosen, as it was the most frequently used analytical method. While other statistical methods were found within the included papers, the use of the Bland–Altman method as the primary outcome metric allowed comparability across the greatest number of studies. This was assessed using the estimates of bias (mean difference between BIA and 4C method) and the limits of agreement (LOAs) as set out by Bland and Altman [11,12]. The LOA provided an estimate of the upper and lower boundaries of the bias between the two methods. The smaller the LOA, the more accurate the estimate of the bias. The upper and lower LOAs are calculated on the mean difference between the 4C model and the BIA comparator (often referred to as bias); in some cases, the LOAs were estimated from the provided plots. Proportional bias, the slope of the regression line taken on the Bland–Altman plot was also considered; ideally, there should be no clinically relevant proportional bias.

A meta-analysis of the Bland–Altman data was deemed inappropriate given the significant heterogeneity of the studies, both with respect to the 4C model methodology and among the BIA devices with respect to machine manufacturer and type. Several authors have suggested guidelines for the use of the Bland–Altman test; these have been evaluated by Gerke et al. [13], with the recommendation to use the checklist of Abu-Arafeh et al. [14]. A modified checklist of Abu-Arafeh et al. [14] was used on the included trials in this review (see Supplementary Table S4).

## 3. Results

**Figure 2:**
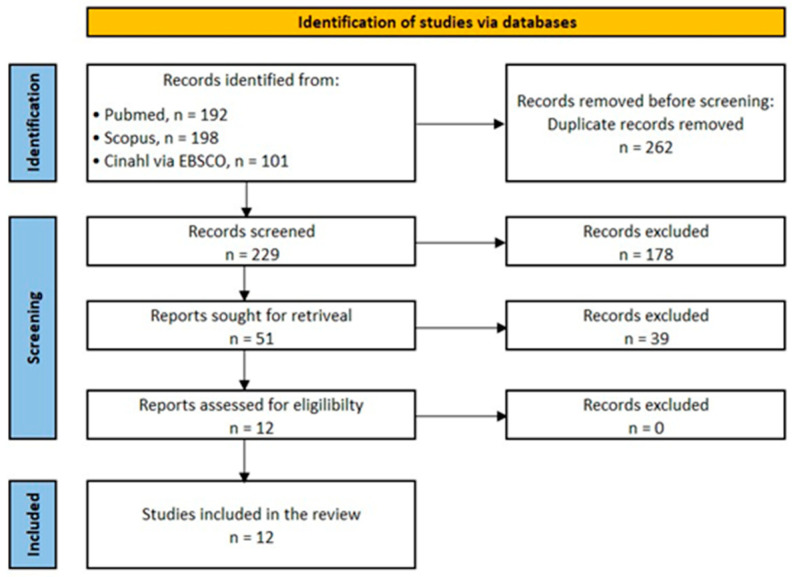
PRISMA flow chart showing the number of articles included or excluded in the review. Additional information on the exclusion of screened papers is available in the Supplementary Information Table S2.

The electronic database search retrieved 491 records, with 262 duplicates subsequently removed, and a further 178 were excluded for not being relevant after reviewing the title and abstract. A total of 51 full-text articles were reviewed for eligibility (Figure 2). In two of these studies [15,16], the X-axis of the Bland–Altman plot contained the 4C model estimate and not the average value of the 4C model and the comparator BIA as specified by Bland–Altman. In one instance, this was a deliberate decision of the authors [16] based on the paper of Krouwer (2008) [17]; however, the argument for not using the average of the two comparator machines on the X-axis value has been disputed [18]. Consequently, a decision was made to exclude these two papers from the analysis to preserve homogeneity with the remaining papers included in the analysis. Therefore, a total of twelve studies that met the eligibility criteria were included in this review (Figure 1). A list of eligible articles and reasons for exclusion of papers is available in Supplementary Table S2.

For this analysis, data for %BF and FFM are reported where possible. While twelve studies were included in this review, some studies included more than one device or provided additional data based on gender or ethnicity; these permutations were treated as separate entries for the purposes of evaluation, giving thirty-four evaluations for percentage body fat from eleven studies, and twenty-nine evaluations for fat-free mass from seven studies.

Jebb et al. [19] investigated two different BIA machines, while Gibson et al. [20] investigated two different BIA models from the same manufacturer (Table 1). Bosy-Westphal et al. [21] tested a BIA machine to develop a prediction equation in a sample of German adults and then applied the equation to a multi-cultural validation sample in the USA (Table 1). Nickerson et al. [22] used one BIA machine while testing four separate body fat equations, providing data on the total sample as well as by sex (Table 1). In a separate study, Nickerson et al. [23] tested one BIA machine and provided an analysis on the total cohort, as well as men and women separately. Blue et al. [24] tested one BIA machine and provided data on different ethnic groups, but the Bland–Altman analysis was conducted only on the total group. Brandner et al. [25] investigated a BIA smartwatch and a multifrequency bioelectrical impedance analysis (MFBIA) machine; only data from the MFBIA machine was included. The Siedler et al. study [26] investigated 15 different BIA machines, most of which were for domestic use; we chose to include just the medical-grade BIA machine (Seca 515/514) that would be used in clinical or research studies. The papers by Gibson et al. [20] and Brewer et al. [27] were included after it was confirmed with the manufacturer that the InBody devices were BIA devices and not BIS devices, as the title of the Gibson paper suggests (email correspondence, 2nd August 2023).

### 3.1. Study Characteristics

Details of the included studies, information on the study populations, the 4C equation used, and the component methodology are provided in Table 1, including the types of BIA machines and their manufacturers.

### 3.2. Study Quality

The quality of the studies, as assessed by the AXIS questionnaire, is presented in Supplementary Table S3a,b. Overall, the quality of the studies was judged as adequate except for sample size justification and questions relating to statistical reporting, as described below. Only two of the included studies, Brandner et al. [25] and Blue et al. [24], provided statistically based sample size calculations, though some justification for the sample size was provided in several cases. With regard to premeasurement restrictions of fluid, alcohol, and strenuous exercise, minimal information was supplied by studies prior to 2013 (Supplementary Table S4).

### 3.3. Statistical Methods

Only studies that used the Bland–Altman agreement analysis for the assessment at the individual level were included in this review. Whilst the Bland–Altman analysis method is straightforward, there is a preferred methodology for using this assessment method. Several guidelines for the use of the Bland–Altman test have been evaluated by Gerke (2020) [13], who recommended the checklist of Abu-Arafeh et al. [14]. A modified checklist of Abu-Arafeh et al. [14] was used on the included trials in this review (see Supplementary Table S5). Assessment of proportional bias through a regression analysis on the plot data points provides additional information as to whether high or low estimates can skew agreement. Some studies reporting using the Bland–Altman method did not supply LOA plots. The Fuller et al. study [28] provided %BF for the 4C model but not the BIA machine, only providing the bias and LOA; some, e.g., Jebb et al. [19], provided only the bias data.

A variety of different statistical methods were reported for random error (precision/reliability) and systematic error or bias reflecting accuracy or validity. Test–retest reliability tests included calculating precision error, using intraclass correlation coefficients (ICC), least-significant change, and the root mean square coefficient of variation. Agreement between devices other than Bland–Altman analysis was assessed by multiple methods, including paired *t*-tests, Pearson correlation coefficient, Deming regression, root mean square error, concordance correlation coefficients (CCC), and equivalence testing using two one-sided *t*-tests (TOST).

### 3.4. 4C Model vs. BIA Results

#### 3.4.1. Bland–Altman—Percentage Body Fat

The summary for the Bland–Altman tests for %BF comparison between BIA and the 4C model is in Table 2. The overall bias between the percentage of body fat recorded by the 4C model and the BIA machine could be considered low, ranging from −2.9% to 4.4% per cent. However, there are wide limits of agreement around the bias estimates in nearly all cases.

#### 3.4.2. Bland–Altman—Fat-Free Mass

The summary for the Bland–Altman tests for FFM comparison between BIA and the 4C model is in Table 3. The bias between FFM recorded by the 4C model and the BIA machine could be considered low, ranging from −3.9 to 1.8 kg; however, there are wide limits of agreement around the bias estimates in all studies.

## 4. Discussion

While BIA machines have been tested against the 4C model numerous times, different criteria have been used to assess the validity of estimates of %BF and FFM from BIA to the 4C model. For our analysis, the use of Bland–Altman limits of agreement was specified as the primary criterion for assessing agreement between a BIA machine and a 4C model, as this was the most used statistical method across studies and allowed for assessment at the individual clinical level. The results of the Bland–Altman analysis comparison in this paper of cross-sectional studies show that at the individual level, estimates of %BF or FFM from the included BIA machines were not adequate estimators of these components compared to the criterion 4C model used (Table 2 and Table 3). The BIA machines tested in this review showed bias estimates ranging from relatively low to high, both in absolute and relative terms (Table 2 and Table 3). On average, the bias for %BF was greater than that for fat-free mass, both in absolute and relative terms. However, these biases were frequently accompanied by wide 95% limits of agreement, reducing their suitability for individual-level assessment of either measure. For clinical interchangeability, even a larger bias can be acceptable if the limits of agreement are narrow and consistently on one side of zero, as this allows for a straightforward correction. In contrast, a small bias with wide limits that cross zero provides little practical value. In those studies, with proportional bias estimates, proportional bias was observed only very occasionally.

Several critical issues need consideration when evaluating the significance of these findings. These are the complexities of the measurements made by the BIA machines and those made by the 4C model, and the method for assessing equivalence between the two methods.

### 4.1. BIA—Validation of Percentage Body Fat and Fat-Free Mass

A diverse array of BIA machines was used by the studies included in this review, differing in several important ways. Firstly, machines could differ in their physical design: from single-frequency, supine, whole-body estimations, using electrodes, to vertical, multiple-frequency, segmental estimations, using foot plates, handles, or handrails; or combinations of these factors (Table 1). Secondly, BIA machines do not directly measure %BF or FFM but rather provide estimates of these components based on validation studies against other body composition techniques used as reference standards. BIA devices may have used different validation methods, which themselves may have needed external validation. For example, if DXA was used as the reference standard, the BIA estimates of %BF or FFM would be limited to the generalization restrictions based on the DXA-specific validation sample characteristics, for example, age, sex, race, body composition, and health status. BIA validity against a 4C model could be confounded by the inherent limitations of the DXA reference model and validation sample. In this case, comparing BIA estimates of %BF and FFM to 4C model estimates may, in fact, be a de facto 4C model assessment of the DXA validation method. BIA machines can use unpublished proprietary regression-based equations in their estimates of TBW, body fat, FFM, or muscle [38]. Information on how these equations are adjusted for race, gender, and age may not be available. Issues can also arise with published equations to generate estimates of %BF or FFM from resistance or reactance data. For example, in the Nickerson et al. paper [22], four different BIA equations were tested in a sample of young men and women; three studies used an RJL device (Deurenberg [35], Chumlea [34], and Sun [37]); the other study used a Xitron 4000B, which is actually a BIS machine (Kyle [36]). The problem with published equations is that different BIA machines do not necessarily give the same raw reactance and resistance measurements [39,40,41]. When using published body composition equations, if the BIA machine being utilized is not using the same BIA machine as, or has not been validated against, the specific machine used in making these equations, then issues of accuracy will occur [42].

While the limitations concerning the accuracy and precision of BIA machines have been discussed [43,44], an often-unexplored issue is the accuracy and precision of the criterion 4C method used itself. Both sides, the comparator and the reference standard, need validation, as they both contribute to the total error observed.

### 4.2. 4C Model—More than One Model

The critical outcome of validation studies is whether an estimate of body composition from one method can be substituted for the same body composition estimate by another method. There are two key factors with respect to measurement instruments: the level of repeatability and accuracy. For a device to have good accuracy, good reliability is very important. Bland and Altman noted that if the criterion method has poor repeatability, then even if the comparator is perfect, the methods will not agree; if both methods have poor repeatability, any comparison will be very problematic [12,45].

If the precision of the 4C model used is unknown, there can be uncertainty as to what degree and on which side of the equation the error lies. There are multiple BIA machines and potential BIA-based equations that could be used to derive estimates of %BF and FFM. There are also multiple 4C model equations available for use, as well as multiple ways to measure them. All 4C equations are derived from first principles; an obvious first consideration is whether all 4C model equations are equivalent or whether there is a preferred 4C model and preferred component methodology.

**Figure 3:**
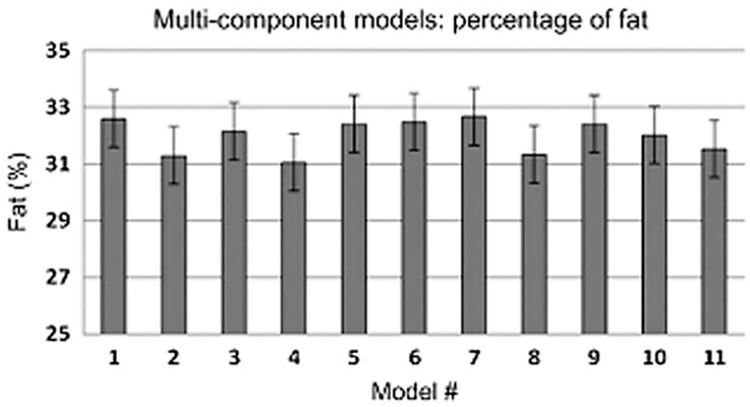
Differences in the estimation of percentage body fat using different 4C model equations [4]. With permission of the journal. Models: 1. Selinger 1977 [31]; 2. Lohman 1986 [46]; 3. Heymsfield et al. 1990 [47] and Baumgartner et al. 1993 [48]; 4. Lohman 1992 [49], Lohman and Going 1993 [50], and Wilson et al. 2012 [7]; 5. Friedl et al. 1992 [51]; 6. Fuller et al. 1992 [28]; 7. Withers et al. 1998 [52] and Heymsfield 1996 [53]; 8. Siconolfi et al. 1995 [54]; 9. Forslund et al. 1996 [55]; 10. Wang et al. 2002 [5]; 11. Wang 2015 [4]. Models 1 (Selinger), 6 (Fuller), and 10 (Wang) were used in the studies selected for this paper. With permission of the journal.

Heymsfield et al. [4] examined the variability in %BF estimates from eleven different 4C model equations using the same raw data obtained from body volume measured by Bod Pod, bone mineral density by iDXA (GE Lunar), and total body water by deuterium dilution. The results showed minimal variation in percentage body fat between the models, ranging from 31.0 ± 1.0 to 32.7 ± 1.0%BF (Figure 3). Although the paper revealed instances of statistically significant proportional bias between models using Bland–Altman analysis, no limits of agreement for the eleven equations were provided. However, without verifying the limits of agreement, even small differences (bias) between methods do not guarantee that one equation can be substituted for another at the individual level. Additionally, there was no comparison of FFM across the eleven equations.

### 4.3. 4C Model—Methods Substitution

**Figure 4:**
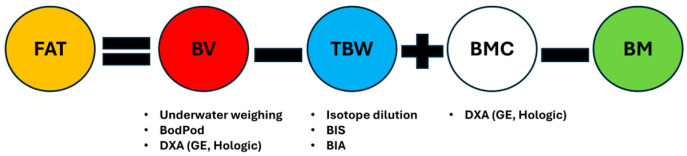
4C model based on Wang 2002 [5]. Note multiple methods for compartment estimation. Fat = fat mass, kg or percentage, BV = body volume, TBW = total body water, BMC = bone mineral content, BM = body mass.

A related second important consideration is the effect of the methodological substitution of a component in a 4C model on estimates of %BF and FFM. While in the study of Heymsfield 2015 [4], all the equations utilized the same raw data obtained using identical component methodologies, this does not necessarily happen between different studies, as shown in Table 1. While the 4C model requires using criterion-based estimation of body volume, bone mineral density, and total body water, the actual methods used for each of these components can vary between studies, as discussed previously. Overall, we can assume there are at least six to twelve possible 4C model iterations, i.e., three body volume * two total body water * one to two bone mineral content for any 4C model equation used (Figure 4). There is also a push for rapid 4C model methodology, using DXA to estimate both body volume and bone mineral content, and BIS to estimate total body water instead of isotope dilution [4,56]. While rapid 4C model methodology may reduce the possible iterations to four, there is a question as to whether some validation against a ‘reference standard’ 4C model is required.

An example of method substitution on the outcomes using the 4C model is with total body water. Total body water can be estimated by isotope dilution or, more recently, for logistical reasons, using the simpler BIS method, which in recent publications has often been obtained using the SFB7 (ImpediMed) device. What would be the effect on the 4C model body composition estimates when SFB7 is used to calculate TBW data instead of isotope dilution?

Blue et al. [24] reported on %BF (and FFM kg) in several ethnic groups using the criterion 4C model of Wang (2002), in which TBW was measured both by deuterium dilution and an SFB7 device. The BIS 4C model estimate of BF% in the various ethnic groups was assigned a Heyward and Wagner rating [57] ranging from very good to excellent-ideal, based on the assessment of the standard error of the estimate (SEE). The Bland–Altman analysis of the same total sample comparing the BIS 4C model estimate of %BF to the criterion 4C model using deuterium dilution showed a bias of −1.5% with LOAs of −5.1% to 2.4%, with a statistically significant proportional bias, R^2^ = 0.1492, *p* < 0.001.

In a study of collegiate athletes, the 5C model of Wang et al. [5] using deuterium dilution to measure total body water (TBW) was compared to two BIS machines, one of which was an SFB7 machine, as well as an MFBIA machine [58]. Firstly, when comparing estimates of TBW per se according to Bland–Altman analysis, the MFBIA machine was the better performer, with a bias of −1.14 L and LOAs of −11.55 to 9.27 L, compared to the SFB7 BIS machine, which had a bias of −1.78 L and LOAs of −16.21 to 12.65 L. Although both devices had very wide LOAs, TOST analysis with 5% equivalence of the TBW estimates found the MFBIA machine was equivalent to the criterion deuterium dilution method, but not the SFB7. Estimates of the 5C model fat mass, using either BIA or SFB7 to estimate TBW in the equation, saw the MFBIA machine having a bias of 0.42 kg and LOAs of −6.85 to 7.69 kg, and the SFB7 BIS machine having a bias of 2.43 kg and LOAs of −18.15 to 23.01 kg. An analysis of fat mass estimates by TOST with 5% equivalence found that none of the machines were equivalent to the criterion method. The authors concluded that the substitution of methods in criterion models could be problematic [58].

In a 4C study in a diverse group of Australian adults, where TBW was once again estimated by deuterium dilution and SFB7, the Bland–Altman analysis between the two techniques gave a bias of 2.53 L and LOAs between −5.92 to 7.07 L, with no proportional bias [59]. When the SFB7 data was substituted into a 4C model (Withers 1998 equation [52]), the bias for fat mass was 1.87 kg with LOAs of −5.16 kg to 4.38 kg, and for fat-free mass, the bias was 1.87 kg, and LOAs were −4.38 kg to 5.16 kg [59].

The most commonly cited paper for SFB7 validation of TBW estimation is that of Moon et al. [60], where the SFB7 was compared to deuterium dioxide in twenty-eight (presumably healthy) young Caucasian men and women aged between 19 and 35 years (mean 24 ± 4 years). For the men, the mean BMI was about 26.0, while for the women, the BMI was low at 20.7. While the bias between SFB7 and deuterium dioxide was small (all subjects −0.09 L, males −0.8 L, females 0.62 L), Bland–Altman analysis of the LOAs was relatively large (all subjects −4.5 to 4.31 L, males −6.11 to 4.49 L, and females −2.20 to 3.45 L). Similar results were seen in another study comparing SFB7 to deuterium dioxide isotope dilution in resistance-trained males, with a bias of only −0.48 L observed, but with LOAs of −5.57 to 5.09 L that were not insignificant [61]. These findings suggest that SFB7 cannot be used in place of isotope dilution for the estimation of TBW at the individual level, at least in ‘healthy’ individuals, in validation studies.

Five of the eleven papers included in this review used BIS to measure total body water; four utilized the SFB7 device, and these four papers were four of the five most recent papers included in this review (Table 4). These results imply that using the SFB7 device in 4C models used in validation studies may introduce a potential source of error, meaning any lack of agreement between the 4C model and the BIA machine used could be in part owing to the 4C model methodology used itself, rather than inherent issues with the BIA machine. The use of the SFB7 device to calculate TBW makes comparison to studies using isotope dilution difficult.

### 4.4. Beyond the 4C Model

Despite the overall poor concordance between the studied BIA machines and the 4C model for estimating %BF and FFM in this review, it is difficult to assess the clinical worth of a BIA machine without evaluating all its critical outputs as discussed.

Owing to these issues, researchers and clinicians have sought to explore the use of the primary outputs of BIA machines, i.e., reactance and resistance, to assess either aspects of body composition or physiological function, such as grip strength or mortality risk [62]. BIA estimates of total body water (TBW), extracellular (ECW), and intracellular water (ICW) can be derived against isotope criterion methods, such as deuterium dilution [63]. The ratio of ECW/TBW and ECW/ICW can also provide useful information on the health status of individuals, with the latter providing particular insight into the health of the muscle component of the body [64].

There is also increasing interest in using another BIA data output, i.e., phase angle. A phase angle provides information on the health of cellular membranes and has been studied with respect to several health outcomes across a range of populations, including muscular health [65,66,67,68,69]. Even here, greater granularity may be needed, as individuals with similar PhA values can have markedly different fluid volumes or %BF [70]. Phase angles can be incorporated into a more in-depth analysis using bioelectrical impedance vector analysis (BIVA) or specific BIVA, though the latter requires the measurement of several body circumferences. Phase angle estimates are again machine-dependent, and the use of BIVA requires population-specific normative data, which is currently limited. However, the BIA International Database project aims to build a multi-ethnic dataset of BIA raw measures using data from multiple countries [71]. This initiative will hopefully provide a substantial normative database for researchers and clinicians.

An additional advantage of many BIA devices is their ability to provide regional body composition estimates, something not quantified by a 4C model. Knowing the size, location, and quality of skeletal muscle, as well as adipose tissue, should be more informative at the individual patient level than whole-body estimates of fat mass and fat-free mass [72]. BIA manufacturers should be encouraged in the evolution of their machines to include additional criterion methods, such as MRI or CT, for total and regional estimates of adipose tissue, skeletal muscle, and muscle quality. There have been large-scale body composition data acquisition projects using MRI, e.g., UK Biobank. MRI can be used to assess skeletal muscle and adipose tissue, both whole body and regionally, and to evaluate muscle ectopic fat deposition.

## 5. Limitations

This systematic review has only dealt with cross-sectional studies in adults designated as healthy, though information on health status was often lacking. This review excluded important population groups, such as athletes or those with chronic disease, given the aim to establish the equivalence between BIA devices and the 4C model in a population with minimal confounding. This review also did not include longitudinal studies. The utility of a body composition device to accurately detect meaningful change either to a treatment intervention or just to monitor disease or health status longitudinally is clinically highly relevant. Most BIA device manufacturers have proprietary equations for their body composition estimates. For many of the included devices in this study, the knowledge regarding their validation methods and cohort demographics is very limited. Premeasurement protocols, which help control for the effects of heavy exercise and water and alcohol intake on hydration status, were inconsistently reported between studies (see Supplementary Table S4). It is difficult to quantify the potential influence of different measurement protocols on the performance of either the BIA device or the 4C model.

## 6. Conclusions

This review compared a combination of BIA machine types and several permutations of the 4C model, varying by equation and methods. The Bland–Altman analysis, with few exceptions, saw low bias coupled with high limits of agreement, indicating acceptable use of BIA at the population level but not at an individual level when using 4C criterion estimates of %BF and FFM. Over the period of the papers selected for this review, there have been significant changes not only in BIA technology, but more recent validation studies have far more precision and validity statistics than earlier papers.

There are several issues concerning both the methodology in BIA machines and that used in 4C model studies, the doubly indirect estimates of %BF and FFM in BIA, and the choice of equation and impact of component substitution in the 4C model. For validation and research applications, isotope-based measures of total body water remain preferable to BIS-derived substitutions. Conceptual issues arise from the use of a more accurate estimation of a 2C model when using the 4C model, while modern body composition assessment requires regional and total estimates of muscle and adipose tissue, as well as an estimate of muscle quality.

Greater emphasis may be placed on raw impedance measures, such as resistance, reactance, and phase angle, which may offer clinically meaningful information independent of proprietary prediction equations. The use of BIA raw data to assess health holds promise. However, the lack of generalizable raw data across BIA machines is a significant shortcoming of this technology. Regardless, the clinical usefulness of BIA should not be defined solely on estimates of fat mass or fat-free mass and needs further improvement to include more comprehensive clinical evidence from future studies.

## Figures and Tables

**Table 1:** The 4-compartment model equation, study demographics, and details of the BIA machine and methods used in the 4-compartment model.

Author, Year, Country	4C Model Equation	*N*	Subject Demographics Health, BMI, Age, Gender, Ethnicity	BIA Model Type	BIA Frequency/Current	BIA Equation Used	BV	TBW/ECW	BMC
FULLER 1992 UK [28]	FULLER 1992 [28]	28	Healthy persons **Men** Age: 38.10 ± 10.7 BMI: 23.40 ± 2.19 Men: 16 **Women** Age: 31.8 ± 11 BMI: 20.86 ± 2.08 Women: 12 Race: Assume largely Caucasian	RJL System BIA-101 Supine Hand-to-foot Whole-body	Frequency: 50 kHz Current: 800 μA	Proprietary equation	Hydrodensitometry Residual lung volume—helium dilution	Isotope Dilution Deuterium Saliva	DXA Lunar DPX-L
JEBB 2000 UK [19]	FULLER 1992 [28]	206	Subjects were healthy, although some were obese **Men** Age: 43.8 ± 16 BMI: 25.9 ± 5.3 Men: 105 Race: **Women** Age: 40.4 ± 13.6 BMI: 25.9 ± 5.5 Women: 101 Race: Assume largely Caucasian	Tanita TBF-305 Vertical Foot-to-foot Whole-body Bodystat-1500 Supine Hand-to-foot Whole-body	Tanita TBF-305 Frequency: 50 kHz Current: 500 µA BodyStat 1500 Frequency: 50 kHz Current: 400 µA According to Meeurwsen 2010 [29]	Tanita Manufacturer equations: **Men** Derived against body density: BD = 1.100696 − 0.107903 × Wt × Z/Ht^2^ + 0.00017 Wt body mass (kg), Ht (m), and Z impedance (Q) Percentage fat calculated from body density: % fat = (4.57/BD − 4.142) × 100 **Women** Prediction equation estimates fat-free mass: FFM = 13.96674 + 0.348613 × Ht^2^/Z + 0.168998 × Wt Percentage fat-(Wt-FFM)/Wt 3 × 100 BodyStat Proprietary equation	Hydrodensitometry Residual lung volume—helium dilution	Isotope Dilution—Deuterium Saliva	DXA Hologic QDR-1000 W “enhanced version of the software to estimate bone mineral (subsequently used to derive ‘ash’), fat and fat-free soft tissue mass”
CHOUINARD 2007 USA [30]	SELINGER 1977 [31]	38	Overweight adults Age: 33.5 ± 7.0 BMI: 27.7 ± 2.0 Men: 7 Women: 38 Race: Caucasian = 36	Tanita TBF-305 Vertical Leg-to-leg Whole-body	Frequency: 50 kHz Current: 500 μA	Tanita Manufacturer equations: **Men** Derived against body density: BD = 1.100696 − 0.107903 × Wt × Z/Ht^2^ + 0.00017 Wt body mass (kg), Ht height (m) and Z impedance (Ohms) Percentage fat calculated from body density: % fat = (4.57/BD − 4.142) × 100 **Women** Prediction equation estimates fat-free mass: FFM = 13.96674 + 0.348613 × Ht^2^/Z + 0.168998 × Wt Percentage fat − (Wt-FFM)/Wt 3 × 100 **Jebb Equation** [19] %FM = −156.1 − 89.1 × ln(Ht) + 45.6 × ln(Wt) + 0.120 × age +0.0494 × Z + [19.6 ln(Ht) (for women] Wt body mass (kg), Ht height (m), Z impedance (Ohms), ln natural log	Hydrodensitometry Residual lung volume—oxygen dilution	Isotope dilution—Tritium ^18^O Urine	DXA Norland XR-36 bone software version 3.7.4/2.1.0
KORTH 2007 Germany [32]	FULLER 1992 [28]	104	Healthy euthyroid weight-stable subjects (non-pregnant or lactating) **Men** Age: 39.1 ± 14.7 BMI: 26.3 ± 3.8 Men: 50 Race: **Women** Age: 35.3 ± 15.4 BMI: 25.5 ± 4.4 Women: 54 Race: ns (assume Caucasian)	Nutri Guard 2000-M analyzer (Data-Input, Frankfurt, Germany) tetrapolar RHS Supine Hand-to-leg	Frequency 50 KHz	TBWmen (l) = 0.396 × (height (cm))^2^/R) +0.143 × weight (kg) + 8:399 TBWwomen (l) = 0.382× (height (cm))^2^/R) + 0.105 × weight (kg) + 8:315 (4) FFM was derived from TBW assuming a water content of 73.2%	Air Displacement Plethysmograph (BODPOD) software version 1.69)	Isotope Dilution—Deuterium Blood	DXA QDR4500A Hologic Inc software (version V8.26a:3)
GIBSON 2008 USA [20]	SELINGER 1977 [31]	146	Apparently healthy men and women **Men** Age: 30.62 ± 13.63 BMI: 27.60 ± 18.76 Men: 75 Race: White 25, Black 25, Hispanic 25 **Women** Age: 33.10 ± 12.90 BMI: 26.30 ± 6.35 Women: 75 Race: White 25, Black 25, Hispanic 25	InBody 720 Vertical Octopolar Whole-body, segmental InBody 320 Vertical Octopolar Whole-body, segmental	InBody 720 Frequency: 1, 5, 50, 250, 500, 1000 kHz Current: 80 µA InBody 320 Frequency: 5, 50, 250 kHz Current: ?	Proprietary equations	Hydrodensitometry Residual lung volume—helium dilution	BIS—4200 HYDRA (Xitron Technologies)	DXA Lunar Prodigy enCORE 2005 software version 9.30.044
BOSY-WESTPHAL 2013 [21]	FULLER 1992 [28]	130	PHASE 1 Age: 40.4 ± 12.2 BMI: 25.0 ± 3.6 Men: 62 Women: 62 Race: Presumably Caucasian PHASE 2 Age: 40.4 ± 12.2 Multi-ethnic validation sample **Men** Age: Caucasian 43.1 ± 15.7; Asian 41.3 ± 14.4; African American 40.9 ± 11.7; Hispanic 39.7 ± 11.7 BMI: Caucasian 26.8 ± 4.6; Asian 23.3 ± 3.5; African American 26.0 ± 3.8; Hispanic 26.7 ± 4.2 Men: Caucasian 16; Asian 18; African American 16; Hispanic 15 **Women** Age: Caucasian 42.7 ± 13.7; Asian 40.7 ± 13.0; African American 40.9 ± 11.7; Hispanic 40.5 ± 13.4 BMI: Caucasian 25.1 ± 34.1; Asian 22.6 ± 1.9; African American 24.6 ± 3.7; Hispanic 27.9 ± 2.9 Women: Caucasian 16; Asian 18; African American 15; Hispanic 16	Sec 515/514 Vertical Octopolar Whole-body, segmental	Frequency: 1, 1.5, 2, 3, 5, 7.5, 10, 15, 20, 30,50, 75, 100, 150, 200, 300, 500, 750, 1000 kHz Current: 100 µA		Air Displacement Plethysmograph (BODPOD)	Isotope Dilution TBW D_2_O ECW NaBr	DXA Hologic Discovery A Hologic Inc software version V12.6.1:3 Lunar iDXA software version 11.4
NICKERSON 2017 USA [22]	WANG 2005 [33]	82	Physically active young and lean Age: 23 ± 5 Ht: 178.2 ± 8.1 Wt: 81.8 ± 13.8 **Men** N = 42 Age: 23 ± 5 Ht: 178.2 ± 8.1 Wt: 81.8 ± 13.8 **Women** N = 40 Age: 22 ± 4 Ht: 164.8 ± 5.6 Wt: 60.3 ± 8.6 Race: NS, assume largely white	Quantum IV Supine Hand-to-foot Whole-body	Frequency: 50 kHz Current: 400A	BIA_CH_ (Chumlea WC 2002) [34] FFM Men = −10:678 + (0.262 × Wt) + (0.652 × Ht^2^/R) + (0:015 × R) FFM Women = −9.529 + (0.168 × Wt) + (0.696 × Ht^2^/R) + (0.016 × R) BIA_DE_ (Deurenberg P 1991) [35] FFM = −12.44 + (0.34 × Ht^2^/R) + (0.1534 × Ht) + (0.273 × Wt) + (0.127 × age) + (4.56 × sex; men = 1; women = 0) BIA_KYLE_ (Kyle UG 2001) [36] FFM Men = −10.68 + (0.65 × Ht^2^/R) + (0.26 × Wt) + (0.02 × R) BIA_SUN_ (Sun SS 2003) [37] FFM Men = −10.68 + (0.65 × Ht^2^/R) + (0.26 × Wt) + (0.02 × R) FFM Women = −9.53 + (0.69 × Ht^2^/R) + (0.17 × Wt) + (0.02 × R) BF% was calculated as BF% = ([BM-FFM]/BM) × 100 R = Resistance, Xc = Reactance, Wt = Weight, Ht = Height	Hydrodensitometry Lung volume—oxygen dilution	BIS (SFB7, Impedimed)	DXA GE Lunar Prodigy software version 14.10.022
BREWER 2021 USA [27]	WANG 2002 [5]	82	Healthy undergraduate students **Men** Age: 19.6 ± 1.1 BMI: 22.1 ± 1.9 Men: 26 **Women** Age: 19.6 ± 1.1 BMI: 22.1 ± 1.8 Women: 56 **Race**: Caucasian n = 66, Asian n = 11, Hispanic n = 3, African American n = 2	InBody 770 Vertical Octopolar Whole-body, segmental	Frequency: 1, 5, 50, 250, 500, 1000 kHz Current: 80 µA	Proprietary Equation	Air Displacement Plethysmography (BodPod; Cosmed, Software Version 5.4.1)	BIS (SFB7, Impedimed)	DXA GE Lunar iDXA enCORE Software version 16
BLUE 2022 USA [24]	WANG 2002 [5]	110	Healthy adults Age: 26.5 ± 6.9 BMI: 25.3 ± 4.0 Gender: 55%(?) **Women** Race: African American/Black n = 22, Caucasian/White n = 22, Asian n = 22, Hispanic n = 22, Native American, n 1 and Multi-racial MR n = 21	InBody 770 Vertical Octapolar Whole-body, segmental	Frequency: 1, 5, 50, 250, 500, 1000 kHz Current: 80 µA	Proprietary Equation	Air Displacement Plethysmography (BodPod; Cosmed,	Isotope Dilution—Deuterium Saliva	DXA GE Lunar iDXA enCORE Software version 16
BRANDNER 2023 USA [25]	WANG 2002 [5]	186	General population **Men** N = 72 Age: 24.85 ± 7.92 BMI: 29.0 ± 7.4 BF%: 22.94 ± 9.66 Race: 49 Caucasian, 21 Black/African-American, 1 Asian, 1 Native American Hispanic 4 **Women** N = 114 Age: 23.11 ± 6.78 BMI: 25.7 ± 5.2 BF%: 32.61 ± 7.64 Race: 78 Caucasian, 31 Black/African-American, 5 Asian, 0 Native-American Hispanic 9	Tanita MC-780 U) MFBIA Vertical Octopolar, Hand-to-hand and foot-to-foot electrodes Whole-body, segmental	Frequency: 5, 50, and 250 kHz Current: Up to 90μA	Proprietary Equation	Lunar iDXA scanner (General Electric, enCORE software v.18.)	BIS (SFB7, Impedimed)	DXA GE Lunar iDXA enCORE software version 18
SIEDLER 2023 USA [26]	WANG 2002 [5]	73	Healthy adults who had maintained a relatively stable body weight Age: 26.2 ± 7.3 BMI: 25.3 ± 4.7 Women: 52% Race: African American/Black = 3, Asian = 10, White/Caucasian = 18, Hispanic = 18, Native Hawaiian/other Pacific Islander = 1	seca mBCA 515/514 Vertical Hand-to-feet Octapolar, Handrail, Whole-body, segmental	Frequency: 1.5, 2, 3, 5, 7.5, 10, 15, 20, 30,50, 75, 100, 150, 200, 300, 500, 750, 1000 kHz Current: 100 µA	Proprietary Equation	Air Displacement Plethysmography BodPod; Cosmed	BIS (SFB7, Impedimed)	DXA GE Lunar Prodigy, enCORE software version 16.2
NICKERSON 2023 USA [23]	WANG 2005 [33]	130	Healthy Hispanic adults 29.2 ± 11.3 BMI: 28.1 ± 5.8 Women: 54%	InBody 570 Vertical Hand-to-feet Tetrapolar Whole-body, segmental	Frequency: 5, 50, and 500 kHz Current: n/a	Proprietary Equation	Air Displacement Plethysmography BodPod; Cosmed	Isotope dilution Deuterium Urine	DXA GE Lunar Prodigy, Encore software version 14.10.022

*N* = sample size, BIA = Bioelectrical Impedance Analysis, BIS = Bioelectrical Impedance Spectroscopy, %BF = percentage body fat, BV = body volume, TBW = total body water, ECW = extracellular water, BMC = bone mineral content. 4C Model Equations: Fuller 1992 [28]. M (kg) = [2.747 × BV − 0.710 × TBW] + [1.460 × BMC − 2.05 × Wt]. FM = fat mass, BV = body volume, BMC = bone mineral content, Wt = body mass. FFM = Body mass − FM. Selinger 1977 [31]. %FM = [[(2.747(BD/ BW)] − [0.714 × (TBW/BW)] + [1.146 × (TBM/BW)] − 2.0503] × 100%, %FM = percentage fat mass; BD = body density, BW = body mass, TBW = total body water; TBM = total body mineral. Wang 2002/2005 [5]/Wang ZM 2005 [33]. FM (kg) = 2.748 × BV − 0.699 × TBW + 1.129 × Mo − 2.051 × BM. FM = fat mass, BV = body volume, Mo = bone mineral, BM = body mass. %BF = (FM/BM) × 100. FFM(kg) = BM − FM.

**Table 2:** Bias, limits of agreement, relative and proportional bias between the BIA machine, and the 4-compartment model for body fat (%).

Author, Year	4C MEAN	4C SD	BIA MEAN	BIA SD	BIAS	BIAS 1 SD	±LOA	Proportional Bias	Relative Bias % Mean 4C
FULLER 1992 [28]	22.28	5.38	n/a	n/a	3.4	3.65	−3.91 to 10.69	No plot	15.2
JEBB 2000 [19] 1. Tanita ALL	n/a	n/a	n/a	n/a	0.9	5.1	−9.1 to 10.9	No plots	n/a
JEBB 2000 [19] 1. Tanita MEN	n/a	n/a	n/a	n/a	−0.9	5.45	−11.58 to 9.78	No plots	n/a
JEBB 2000 [19] 1. Tanita WOMEN	n/a	n/a	n/a	n/a	2.7	4.05	−5.24 to 10.64	No plots	n/a
JEBB 2000 [19] 2. BodyStat ALL	n/a	n/a	n/a	n/a	−1.5	4.65	−10.61 to 7.61	No plots	n/a
JEBB 2000 [19] 2. BodyStat MEN	n/a	n/a	n/a	n/a	−1.5	4.9	−11.1 to 8.1	No plots n/a—bias	n/a
JEBB 2000 [19] 2. BodyStat WOMEN	n/a	n/a	n/a	n/a	−1.4	4.35	−9.93 to 7.13	No plots n/a—bias	n/a
CHOUINARD 2007 [30] Tanita Eq	34.8	6.4	35.1	6.1	0.3	2.17	−3.92 to 4.58	r = −0.15 *p* =0.370	1.0
CHOUINARD 2007 [30] Jebb Eq	34.8	6.4	34.4	5.6	0.4	2.28	−4.07 to 4.87	No plot	1.2
GIBSON 2008 [20] INBODY 720 MEN	21.34	9.25	20.98	8.85	0.23	?	~ −10.1 to 10.1	r = 0.1395 *p* > 0.05	1.2
GIBSON 2008 [20] INBODY 720 WOMEN	35.04	9.01	32.05	9.82	3.0	?	~ −9 to 9	r = 0.1803 *p* > 0.05	8.5
GIBSON 2008 [20] INBODY 320 MEN	21.34	9.25	21.02	8.56	0.20	?	~−10.1 to 10.1	r = 0.1395 *p* > 0.05	0.9
GIBSON 2008 [20] INBODY 320 WOMEN	35.04	9.01	32.55	9.55	2.5	?	~ −9 to 9	r = 0.1277 *p* > 0.05	7.1
NICKERSON 2017 [22] CHUMLEA EQ ALL	21.34	7.8	24.9	7.6	3.1	3.0	−2.8 to 9.0	−0.06	14.5
NICKERSON 2017 [22] CHUMLEA EQ MEN	16.7	6.2	19.7	5.5	3.0	2.7	−2.3 to 8.2	−0.28	18.0
NICKERSON 2017 [22] CHUMLEA EQ WOMEN	27.2	5.3	30.4	5.3	3.2	3.3	−3.3 to 9.7	0.08	11.8
NICKERSON 2017 [22] DEURENBURG EQ ALL	21.8	7.8	25.5	6.9	3.7	3.8	−3.8 to 11.1	−0.26 (*p* ≤ 0.05)	17.0
NICKERSON 2017 [22] DEURENBURG EQ MEN	16.7	6.2	21.2	5.9	4.4	4.0	−3.6 to 12.4	−0.09	26.3
NICKERSON 2017 [22] DEURENBURG EQ WOMEN	27.2	5.3	30.1	4.4	2.9	3.25	−3.6 to 9.4	−0.29	10.7
NICKERSON 2017 [22] KYLE EQ ALL	21.8	7.8	24.1	7.2	2.3	3.7	−4.3 to 8.8	−0.20	10.6
NICKERSON 2017 [22] KYLE EQ MEN	16.7	6.2	19.6	5.8	2.9	3.30	−3.8 to 9.5	−0.12	17.4
NICKERSON 2017 [22] KYLE EQ WOMEN	27.2	5.3	28.8	5.1	1.6	3.15	−4.7 to 7.9	−0.07	5.9
NICKERSON 2017 [22] SUN EQ ALL	21.8	7.8	21.7	7.3	−0.1	3.2	−6.5 to 6.2	−0.16	−0.46
NICKERSON 2017 [22] SUN EQ MEN	16.7	6.2	17.3	5.7	0.6	2.85	−5.1 to 6.2	−0.19	3.66
NICKERSON 2017 [22] SUN EQ WOMEN	27.2	5.3	26.3	5.8	−0.9	3.4	−7.7 to 5.9	0.15	−3.3
BREWER 2021 CS [27] ALL	20.7	8.2	22.3	7.5	1.63 Plot works only if bias is −1.63	3.85 Calculated from SEE	−9.3 to 6.0	R^2^ = 0.0324	n/a
BREWER 2021CS [27] MEN	12.0	4.4	13.4	3.6	1.4 Plot works only if bias is −1.4	2.31 Calculated from SEE	~−5.9 to 5.9	R^2^ = 0.1308	n/a
BREWER 2021 CS WOMEN [27]	24.8	6.2	26.5	4.7	1.7 Plot works only if bias is −1.73	4.72 Calculated from SEE	−11.0 to 7.5	R^2^ = 0.1242	n/a
BLUE 2022 [24] INBODY 770 Total sample	Asian 24.3 Black 26.4 White 24.5 Hispanic 27.2 Multi-Racial 25.1	Asian 6.6 Black 9.5 White 10.6 Hispanic 8.4 Multi-Racial 11.7	Asian 23.7 Black 26.9 White 23.2 Hispanic 26.8 Multi-Racial 25.1	Asian 6.3 Black 9.7 White 9.8 Hispanic 8.3 Multi-Racial 11.3	−0.4	2.91	−6.0 to 5.3	R^2^ = 0.0053 *p* = 0.449	n/a
BRANDNER 2023 [25]	28.9	9.7	26.6	8.4	−2.9	4.4	−11.52 to 5.72	R^2^ = 0.06 *p* < 0.001	−10.0
SIEDLER 2023 [26] Seca 515/514	24.7	9.1	24.5	9.2	−0.2		−6.61 to 6.21	Slope 0.02 *p* ≥ 0.05	−0.8
NICKERSON 2023 [23] InBody 570 ALL	31.9	10.1	32.4	10.1	0.5	2.65	−4.79 to 5.79	R^2^ = 0.00	1.6
NICKERSON 2023 [23] InBody 570 MEN	26.4	8.8	27.9	9.0	0.2	2.25	−4.2 to 4.6	R^2^ = 0.02	0.76
NICKERSON 2023 [23] InBody 570 WOMEN	36.7	8.6	37.1	8.5	0.1	1.84	−3.5 t0 3.7	R^2^ = −0.01	0.27

n/a = not available, ? = unable to compute.

**Table 3:** Bias, limits of agreement, relative and proportional bias between the BIA machine, and the 4-compartment model for fat-free mass (kg).

Author, Year	4C Mean	4C SD	BIA Mean	BIA SD	BIAS	1 SD	LOA	Proportional Bias	Relative Bias % 4C Mean
FULLER 1992 [28]	51.61	10.24	n/a	n/a	−2.4	3.02	−8.35 to 3.49	n/d	−4.7
KORTH 2007 [32] ALL	57.3	11.5	57.0	11.3	+0.4	4.12	−7.59 to 8.33	r = 0.06 *p* > 0.05 No plots	0.6
KORTH 2007 [32] MEN	66.7	7.6	66.8	7.0	−0.1	4.88	−9.67 to 9.45	r = 0.13 *p* > 0.05 No plots	−0.2
KORTH 2007 [32] WOMEN	48.7	6.7	47.9	5.1	+0.8	3.09	−5.25 to 6.87	r = 0.55 *p* < 0.001 No plots	1.7
BOSY-WESTPHAL 2013 [21] Prediction	ALL 55.4 MALE 65.2 FEMALE 45.4	12.0 7.4 5.8	55.4 65.4 45.4	11.8 6.6 6.0	−0.05 from plot	1.90 from plot	−3.8 to 3.7	n/a	−0.1
BOSY-WESTPHAL 2013 [21] Validation-ALL	51.8	10.3	51.0	10.5	0.8	1.9	−2.92 to 4.52	n/a	1.5
BOSY-WESTPHAL 2013 [21] Validation-WHITE	53.1	10.8	52.4	10.3	0.7	2.1	−3.42 to 4.82	n/a	1.3
BOSY-WESTPHAL 2013 [21] Validation-ASIAN	47.8	8.7	47.1	9.9	0.7	1.8	−2.83 to 4.23	n/a	1.5
BOSY-WESTPHAL 2013 [21] Validation-BLACK	57.2	11.0	55.7	11.4	1.5	1.7	−1.83 to 4.83	n/a	2.7
BOSY-WESTPHAL 2013 [21] Validation-HISPANIC	50.0	8.5	49.6	8.8	0.4	1.8	−3.13 to 3.93	n/a	0.8
NICKERSON 2017 [22] CHUMLEA EQ ALL	56.0	14.4	53.7	13.9	−2.2	2.1	−6.4 to 2.0	−0.26	−3.9
NICKERSON 2017 [22] CHUMLEA EQ MEN	67.7	9.7	65.3	9.1	−2.4	2.2	−6.8 to 1.90	−0.26	−3.5
NICKERSON 2017 [22] CHUMLEA EQ WOMEN	43.6	5.4	41.6	4.1	−2.1	2.05	−6.1 to 2.0	−0.62	−4.8
NICKERSON 2017 [22] DEURENBURG EQ ALL	56.0	14.4	53.1	12.5	−2.9	3.1	−9.1 to 3.3	−0.62	−5.2
NICKERSON 2017 [22] DEURENBURG EQ MEN	67.7	9.7	63.8	7.0	−3.9	3.6	−11.1 to 3.3	−0.76	−5.8
NICKERSON 2017 [22] DEURENBURG EQ WOMEN	43.6	5.4	41.8	4.2	−1.8	2.1	−6.0 to 2.3	−0.57	−4.1
NICKERSON 2017 [22] KYLE EQ ALL	56.0	14.4	54.1	12.8	−2.0	2.6	−7.0 to 3.3	−0.61	−3.5
NICKERSON 2017 [22] KYLE EQ MEN	67.7	9.7	65.1	7.4	−2.6	2.9	−8.4 to 3.2	−0.78	−3.8
NICKERSON 2017 [22] KYLE EQ WOMEN	43.6	5.4	42.5	3.9	−1.8	2.0	−5.1 to 2.9	−0.73	−4.1
NICKERSON 2017 [22] SUN EQ ALL	56.0	14.4	55.9	13.6	−0.9	2.2	−4.5 to 4.3	−0.39	−1.6
NICKERSON 2017 [22] SUN EQ MEN	67.7	9.7	67.2	8.9	−0.5	2.3	−5.1 to 4.1	−0.33	−0.7
NICKERSON 2017 [22] SUN EQ WOMEN	43.6	5.4	44.0	3.9	0.3	2.0	−3.7 to 4.4	−0.75	0.7
BREWER 2021 [27] ALL	50.1	9.7	49.1	9.6	−0.97 Plot works only if the bias is 0.97	2.18 Calculated from SEE	−5.2 to 3.3	R^2^ = 0.002 *p* < 0.001	n/a
BREWER 2021 [27] MEN	61.7	7.8	60.7	7.4	−1.0	1.64 Calculated from SEE	−4.2 to 2.2 Plot mix-up in the paper	R^2^ = 0.0736	1.6
BREWER 2021 [27] WOMEN	44.7	4.3	43.8	4.3	−0.97 Plot works only if the bias is 0.97	2.3 Calculated from SEE	−3.6 to 5.5	R^2^ = 0.0006	3.27
BRANDNER 2023 [27]	55.0	14.2	56.8	12.3	1.8	3.2	−4.47 to 8.07	β = −0.14 Intercept: 5.64 R^2^ = 0.25 *p* < 0.001	n/a
NICKERSON 2023 [27] InBody 570 ALL	52.5	10.8	52.0	10.7	0.4	2.04	−3.5 to 4.32	R^2^ = 0.01	0.76
NICKERSON 2023 [27] InBody 570 MEN	61.7	7.5	61.2	7.4	0.5	2.25	−3.9 to 4.9	R^2^ = −0.01	0.81
NICKERSON 2023 [27] InBody 570 WOMEN	44.5	5.6	44.2	5.6	0.3	1.84	−3.3 to 3.9	R^2^ = −0.01	0.67

BIA = Bioelectrical impedance Analysis, SD = standard deviation, LOA = limits of agreement, n/a—not available.

**Table 4:** Bias and limits of agreement for studies that used SFB7 BIS for measurement of total body water in their 4C equation. All studies used the Wang 4C equation.

Author, Year	4C MEAN	4C SD	BIA MEAN	BIA SD	BIAS	BIAS 1 SD	±LOA
NICKERSON 2017 [22] CHUMLEA EQ ALL	21.34	7.8	24.9	7.6	3.1	3.0	−2.8 to 9.0
NICKERSON 2017 [22] CHUMLEA EQ MEN	16.7	6.2	19.7	5.5	3.0	2.7	−2.3 to 8.2
NICKERSON 2017 [22] CHUMLEA EQ WOMEN	27.2	5.3	30.4	5.3	3.2	3.3	−3.3 to 9.7
NICKERSON 2017 [22] DEURENBURG EQ ALL	21.8	7.8	25.5	6.9	3.7	3.8	−3.8 to 11.1
NICKERSON 2017 [22] DEURENBURG EQ MEN	16.7	6.2	21.2	5.9	4.4	4.0	−3.6 to 12.4
NICKERSON 2017 [22] DEURENBURG EQ WOMEN	27.2	5.3	30.1	4.4	2.9	3.25	−3.6 to 9.4
NICKERSON 2017 [22] KYLE EQ ALL	21.8	7.8	24.1	7.2	2.3	3.7	−4.3 to 8.8
NICKERSON 2017 [22] KYLE EQ MEN	16.7	6.2	19.6	5.8	2.9	3.30	−3.8 to 9.5
NICKERSON 2017 [22] KYLE EQ WOMEN	27.2	5.3	28.8	5.1	1.6	3.15	−4.7 to 7.9
NICKERSON 2017 [22] SUN EQ ALL	21.8	7.8	21.7	7.3	−0.1	3.2	−6.5 to 6.2
NICKERSON 2017 [22] SUN EQ MEN	16.7	6.2	17.3	5.7	0.6	2.85	−5.1 to 6.2
NICKERSON 2017 [22] SUN EQ WOMEN	27.2	5.3	26.3	5.8	−0.9	3.4	−7.7 to 5.9
BREWER 2021 CS [27] ALL	n/a	n/a	n/a	n/a	1.63	~3.85 Approx from SEE	~−5.9 to 9.2 Approx from SEE and plot
BREWER 2021 CS [27] MEN	n/a	n/a	n/a	n/a	1.4	~2.2	~−2.9 to 5.7 Approx from SEE
BREWER 2021 CS [27] WOMEN	n/a	n/a	n/a	n/a	1.73	~4.4	~−6.9 to 10.4 Approx from SEE
BRANDNER 2023 [25]	28.9	9.7	26.6	8.4	−2.9	4.4	−11.52 to 5.72
SIEDLER 2023 [26] Seca 515/514	24.7	9.1	24.5	9.2	−0.2	0.41	−1.0 to 0.6

BIA = Bioelectrical Impedance Analysis, SD = standard deviation, LOA = limits of agreement. n/a—not available.

## Data Availability

No new data were created or analyzed in this study. Data sharing is not applicable to this article.

## Supplementary Materials

The following supporting information can be downloaded at https://www.mdpi.com/article/10.3390/jfmk11010065/s1: Table S1. Systematic review search criteria. Table S2. Eligible articles (n = 49) and reasons for exclusion. Table S3a. Quality of assessment of included studies by Appraisal tool for Cross-Sectional Studies, Questions 1 to 11 (AXIS). Table S3b. Quality of assessment of included studies by Appraisal tool for Cross-Sectional Studies Questions 12 to 20 (AXIS). Table S4. Stated pre-measurement study protocols. Table S5. Abbreviated and modified Abu-Arafeh Bland-Altman Checklist on included studies. Table S6. Summary of methodological heterogeneity across included studies.
