# Supplementary material for: The Validity of Bioelectrical Impedance Analysis Compared to a Four-Compartment Model in Healthy Adults: A Systematic Review
Source: J Funct Morphol Kinesiol. 2026 Jan 31;11(1):65. doi: 10.3390/jfmk11010065 (PMC12922097; doi:10.3390/jfmk11010065)
Supplement: Supplementary file 1 [file jfmk-11-00065-s001.zip › jfmk-4064109-supplementary.pdf]

SUPPLEMENTAL INFORMATION

The search string was designed for PubMed and translated for use in other databases using the Polyglot Search Translator [1].

Supplemental Table S1. Systematic review search criteria

**PUBMED 20FEB23**	("Electric Impedance"[Mesh] OR Bioimpedance[TIAB] OR "bioelectrical impedance"[TIAB] OR "electric impedance"[TIAB] OR "electrical impedance"[TIAB] OR "bioelectrical impedance spectroscopy"[TIAB] OR "bioimpedance spectroscopy"[TIAB] OR Impedance[TIAB] OR "Electric Resistance"[TIAB] OR "Electrical Resistance"[TIAB] OR "Ohmic Resistance*"[TIAB] OR "Bioelectrical Impedance"[TIAB] OR "Biolectric Impedance") **AND** ("Muscle, Skeletal"[Mesh] OR “Body Composition”[Mesh] OR “Body composition*”[TIAB] OR “body fat”[TIAB] OR “adipose tissue”[MeSH] OR “fat mass”[TIAB] OR “fat free mass”[TIAB] OR “fat-free mass”[TIAB] OR “skeletal muscle”[TIAB] OR “muscle mass”[TIAB] OR “lean mass”[TIAB] OR “lean body mass”) **AND** ("4C Model"[TIAB] OR 4C-Model[TIAB] OR "Four Compartment"[TIAB] OR Four-Component[TIAB] OR “4-Compartment”[TIAB] OR “4-Component”[TIAB] OR Multicomponent[TIAB] OR Multi-Component[TIAB] OR Multicompartment[TIAB] OR Multi-Compartment [TIAB])	N=173
**SCOPUS 20FEB23**	(INDEXTERMS("Electric Impedance") OR TITLE-ABS("Bioimpedance") OR TITLE-ABS("bioelectrical impedance") OR TITLE-ABS("electric impedance") OR TITLE-ABS("electrical impedance") OR TITLE-ABS("bioelectrical impedance spectroscopy") OR TITLE-ABS("bioimpedance spectroscopy") OR TITLE-ABS("Impedance") OR TITLE-ABS("Electric Resistance") OR TITLE-ABS("Electrical Resistance") OR TITLE-ABS("Ohmic Resistance*") OR TITLE-ABS("Bioelectrical Impedance") OR "Biolectric Impedance") AND (INDEXTERMS("Muscle, Skeletal") OR INDEXTERMS("Body Composition") OR TITLE-ABS("Body composition*") OR TITLE-ABS("body fat") OR INDEXTERMS("adipose tissue") OR TITLE-ABS("fat mass") OR TITLE-ABS("fat free mass") OR TITLE-ABS("fat-free mass") OR TITLE-ABS("skeletal muscle") OR TITLE-ABS("muscle mass") OR TITLE-ABS("lean mass") OR "lean body mass") AND (TITLE-ABS("4C Model") OR TITLE-ABS("4C-Model") OR TITLE-ABS("Four Compartment") OR TITLE-ABS("Four-Component") OR TITLE-ABS("4-Compartment") OR TITLE-ABS("4-Component") OR TITLE-ABS("Multicomponent") OR TITLE-ABS("Multi-Component") OR TITLE-ABS("Multicompartment") OR TITLE-ABS("Multi-Compartment"))	N=175
**CINAHL via ebsco 20FEB23**	((MH "Electric Impedance+") OR (TI Bioimpedance OR AB Bioimpedance) OR (TI "bioelectrical impedance" OR AB "bioelectrical impedance") OR (TI "electric impedance" OR AB "electric impedance") OR (TI "electrical impedance" OR AB "electrical impedance") OR (TI "bioelectrical impedance spectroscopy" OR AB "bioelectrical impedance spectroscopy") OR (TI "bioimpedance spectroscopy" OR AB "bioimpedance spectroscopy") OR (TI Impedance OR AB Impedance) OR (TI "Electric Resistance" OR AB "Electric Resistance") OR (TI "Electrical Resistance" OR AB "Electrical Resistance") OR (TI "Ohmic Resistance*" OR AB "Ohmic Resistance*") OR (TI "Bioelectrical Impedance" OR AB "Bioelectrical Impedance") OR "Biolectric Impedance") AND ((MH "Muscle, Skeletal+") OR (MH "Body Composition+") OR (TI "Body composition*" OR AB "Body composition*") OR (TI "body fat" OR AB "body fat") OR (MH "adipose tissue+") OR (TI "fat mass" OR AB "fat mass") OR (TI "fat free mass" OR AB "fat free mass") OR (TI "fat-free mass" OR AB "fat-free mass") OR (TI "skeletal muscle" OR AB "skeletal muscle") OR (TI "muscle mass" OR AB "muscle mass") OR (TI "lean mass" OR AB "lean mass") OR "lean body mass") AND ((TI "4C Model" OR AB "4C Model") OR (TI 4C-Model OR AB 4C-Model) OR (TI "Four Compartment" OR AB "Four Compartment") OR (TI Four-Component OR AB Four-Component) OR (TI 4-Compartment OR AB 4-Compartment) OR (TI 4-Component OR AB 4-Component) OR (TI Multicomponent OR AB Multicomponent) OR (TI Multi-Component OR AB Multi-Component) OR (TI Multicompartment OR AB Multicompartment) OR (TI Multi-Compartment OR AB Multi-Compartment))	N=96

Supplemental Table S2 Eligible articles (n=49) and reasons for exclusion

Guo SM, Roche AF and Houtkooper L. **Fat-free mass in children and young adults predicted from bioelectric impedance and anthropometric variables**. Am J Clin Nutr. 1989; 50 (3):435-43.	Full text n=49 Pubmed eligible - **no Bland Altman analysis**
Fuller NJ, Jebb SA, Laskey MA, Coward WA and Elia M. **Four-component model for the assessment of body composition in humans: comparison with alternative methods, and evaluation of the density and hydration of fat-free mass**. Clin Sci (Lond). 1992; 82 (6):687-93.	Full text n=49 Pubmed eligible - included
Forslund AH, Johansson AG, Sjödin A, Bryding G, Ljunghall S*, et al.* **Evaluation of modified multicompartment models to calculate body composition in healthy males**. Am J Clin Nutr. 1996; 63 (6):856-62.	Full text n=49 Pubmed eligible but excluded - TBW measured by BIA not BIS or isotopes
Fogelholm M and Van Marken Lichtenbelt W. **Comparison of body composition methods: a literature analysis**. Eur J Clin Nutr. 1997; 51 (8):495-503.	Full text n=49 Pubmed eligible but excluded – literature review
Goran MI, Toth MJ and Poehlman ET. **Cross-validation of anthropometric and bioelectrical resistance prediction equations for body composition in older people using the 4-compartment model as a criterion method**. J Am Geriatr Soc. 1997; 45 (7):837-843.	Full text n=49 Pubmed eligible – excluded, used one equation that was validated on 4C model - required thigh circumference though
Fogelholm GM, Sievänen HT, Van Marken Lichtenbelt WD and Westerterp KR. **Assessment of fat-mass loss during weight reduction in obese women**. Metabolism. 1997; 46 (8):968-75.	Full text n=49 Pubmed eligible – excluded as could not compute limits of agreement for percentage body fat or fat-free mass
Wang ZM, Deurenberg P, Guo SS, Pietrobelli A, Wang J*, et al.* **Six-compartment body composition model: inter-method comparisons of total body fat measurement**. Int J Obes Relat Metab Disord. 1998; 22 (4):329-37.	Full text n=49 Pubmed eligible but excluded - not 4C v BIA
Evans EM, Saunders MJ, Spano MA, Arngrimsson SA, Lewis RD, *et al.* **Body-composition changes with diet and exercise in obese women: a comparison of estimates from clinical methods and a 4-component model**. Am J Clin Nutr. 1999; 70 5-12.	Full text n=49 Pubmed eligible - excluded, change data
Jebb SA, Cole TJ, Doman D, Murgatroyd PR and Prentice AM. **Evaluation of the novel Tanita body-fat analyser to measure body composition by comparison with a four-compartment model**. Br J Nutr. 2000; 83 (2):115-22.	Full text n=49 Pubmed eligible - included
Evans EM, Arngrimsson SA and Cureton KJ. **Body composition estimates from multicomponent models using BIA to determine body water**. Med Sci Sports Exerc. 2001; 33 (5):839-45.	Full text n=49 Pubmed eligible but excluded - just used BIA instead of isotopes not 4C vs BIA
Deurenberg P and Deurenberg-Yap M. **Validation of skinfold thickness and hand-held impedance measurements for estimation of body fat percentage among Singaporean Chinese, Malay and Indian subjects**. Asia Pac J Clin Nutr. 2002; 11 (1):1-7.	Full text n=49 Pubmed eligible – excluded, Bland-Altman plot x-axis used 4C model estimate and not the average value of the 4C model and the comparator BIA as specified by Bland-Altman
Deurenberg P, Deurenberg-Yap M and Schouten FJ. **Validity of total and segmental impedance measurements for prediction of body composition across ethnic population groups**. Eur J Clin Nutr. 2002; 56 (3):214-20.	Full text n=49 Pubmed eligible – excluded, out of scope

Sun SS, Chumlea WC, Heymsfield SB, Lukaski HC, Schoeller D, *et al.* **Development of bioelectrical impedance analysis prediction equations for body composition with the use of a multicomponent model for use in epidemiologic surveys**. Am J Clin Nutr. 2003; 77 (2):331-340.	Full text n=49 Pubmed eligible but excluded - derivation of FFM and TBW equations using BIA rather than comparison between 4C model and BIA.
Bartok C, Schoeller DA, Randall Clark R, Sullivan JC and Landry GL. **The effect of dehydration on wrestling minimum weight assessment**. Med Sci Sports Exerc. 2004; 36 (1):160-7.	Full text n=49 Pubmed eligible - excluded, sport, dehydration
Clark RR, Bartok C, Sullivan JC and Schoeller DA. **Minimum weight prediction methods cross-validated by the four-component model**. Med Sci Sports Exerc. 2004; 36 (4):639-47.	Full text n=49 Pubmed eligible but excluded, sport, minimum weight
Clark RR, Bartok C, Sullivan JC and Schoeller DA. **Is leg-to-leg BIA valid for predicting minimum weight in wrestlers?** Med Sci Sports Exerc. 2005; 37 (6):1061-8.	Full text n=49 Pubmed eligible - excluded, sport
Jebb SA, Siervo M, Murgatroyd PR, Evans S, Fruhbeck G, *et al.* **Validity of the leg-to-leg bioimpedance to estimate changes in body fat during weight loss and regain in overweight women: a comparison with multi-compartment models**. Int J Obes (Lond). 2007; 31 (5):756-762.	Full text n=49 Pubmed eligible - excluded, no percentage body fat
Korth O, Bosy-Westphal A, Zschoche P, Glüer CC, Heller M, *et al.* **Influence of methods used in body composition analysis on the prediction of resting energy expenditure**. Eur J Clin Nutr. 2007; 61 (5):582-9.	Full text n=49 Pubmed eligible - included
Chouinard LE, Schoeller DA, Watras AC, Clark RR, Close RN, *et al.* **Bioelectrical impedance vs. four-compartment model to assess body fat change in overweight adults**. Obesity (Silver Spring). 2007; 15 (1):85-92.	Full text n=49 Pubmed eligible - included , baseline only
Gibson AL, Holmes JC, Desautels RL, Edmonds LB and Nuudi L. **Ability of new octapolar bioimpedance spectroscopy analyzers to predict 4-component-model percentage body fat in Hispanic, black, and white adults**. Am J Clin Nutr. 2008; 87 (2):332-338.	Full text n=49 Pubmed eligible - included
Laforgia J, Gunn S and Withers RT. **Body composition: validity of segmental bioelectrical impedance analysis**. Asia Pac J Clin Nutr. 2008; 17 (4):586-91.	Full text n=49 Pubmed eligible - excluded, **no Bland-Altman**
Minderico CS, Silva AM, Keller K, Branco TL, Martins SS, *et al.* **Usefulness of different techniques for measuring body composition changes during weight loss in overweight and obese women**. Br J Nutr. 2008; 99 (2):432-41.	Full text n=49 Pubmed eligible - excluded insufficient baseline data
Wilson JP, Strauss BJ, Fan B, Duewer FW and Shepherd JA. **Improved 4-compartment body-composition model for a clinically accessible measure of total body protein**. Am J Clin Nutr. 2013; 97 (3):497-504.	Full text n=49 Pubmed eligible but excluded - not 4C v BIA
Bosy-Westphal A, Schautz B, Later W, Kehayias JJ, Gallagher D, *et al.* **What makes a BIA equation unique? Validity of eight-electrode multifrequency BIA to estimate body composition in a healthy adult population**. Eur J Clin Nutr. 2013; 67 Suppl 1 S14-S21.	Full text n=49 Pubmed eligible – included, prediction equation,
Moon JR, Stout JR, Smith-Ryan AE, Kendall KL, Fukuda DH, *et al.* **Tracking fat-free mass changes in elderly men and women using single-frequency bioimpedance and dual-energy**	Full text n=49 Pubmed eligible but excluded - used BIS (SBF7) instead of BIA

**X-ray absorptiometry: a four-compartment model comparison**. Eur J Clin Nutr. 2013; 67 Suppl 1 S40-6.	
Pourhassan M, Schautz B, Braun W, Gluer CC, Bosy-Westphal A, *et al.* **Impact of body-composition methodology on the composition of weight loss and weight gain**. Eur J Clin Nutr. 2013; 67 (5):446-54.	Full text n=49 Pubmed eligible but excluded - not 4C v BIA
Kuriyan R, Thomas T, Ashok S, Jayakumar J and Kurpad AV. **A 4-compartment model based validation of air displacement plethysmography, dual energy X-ray absorptiometry, skinfold technique & bio-electrical impedance for measuring body fat in Indian adults**. Indian J Med Res. 2014; 139 (5):700-707.	Full text n=49 Pubmed eligible - excluded, Bland-Altman plot x-axis used 4C model estimate and not the average value of the 4C model and the comparator BIA as specified by Bland-Altman
Marshall NE, Murphy EJ, King JC, Haas EK, Lim JY, *et al.* C**omparison of multiple methods to measure maternal fat mass in late gestation.** Am J Clin Nutr. 2016; 103 (4):1055-63.	Full text n=49 Pubmed eligible but excluded pregnancy
Nickerson BS, Esco MR, Bishop PA, Schumacker RE, Richardson MT, *et al.* **Validity of selected bioimpedance equations for estimating body composition in men and women: A four-compartment model comparison.** J Strength Cond Res. 2017; 31 (7):1963-1972.	Full text n=49 Pubmed eligible - included equations
Ng BK, Liu YE, Wang W, Kelly TL, Wilson KE, *et al.* V**alidation of rapid 4-component body composition assessment with the use of dual-energy X-ray absorptiometry and bioelectrical impedance analysis.** Am J Clin Nutr. 2018; 108 (4):708-715.	Full text n=49 Pubmed eligible - **excluded, used linear regression but not Bland-Altman**
Nickerson BS and Tinsley GM. **Utilization of BIA-Derived Bone Mineral Estimates Exerts Minimal Impact on Body Fat Estimates via Multicompartment Models in Physically Active Adults.** J Clin Densitom. 2018; 21 (4):541-549.	Full text n=49 Pubmed eligible - excluded 5C was criterion model
Kondo E, Sagayama H, Yamada Y, Shiose K, Osawa T, *et al.* E**nergy Deficit Required for Rapid Weight Loss in Elite Collegiate Wrestlers.** Nutrients. 2018; 10 (5):	Full text n=49 Pubmed eligible but excluded, sport, no direct comparison
Fedewa MV, Nickerson BS and Esco MR. A**ssociations of body adiposity index, waist circumference, and body mass index in young adults.** Clin Nutr. 2019; 38 (2):715-720.	Full text n=49 Pubmed eligible but excluded - no comparison made to BIA
Domingos C, Matias CN, Cyrino ES, Sardinha LB and Silva AM. T**he usefulness of Tanita TBF-310 for body composition assessment in Judo athletes using a four-compartment molecular model as the reference method.** Rev Assoc Med Bras (1992). 2019; 65 (10):1283-1289.	Full text n=49 Pubmed eligible - excluded, athletes
Jensen B, Braun W, Geisler C, Both M, Kluckmann K, *et al.* **Limitations of fat-free mass for the assessment of muscle mass in obesity.** Obes Facts. 2019; 12 (3):307-315.	Full text n=49 Pubmed eligible but **excluded, no Bland Altman analysis**
Graybeal AJ, Moore ML, Cruz MR and Tinsley GM. B**ody composition assessment in male and female bodybuilders: A 4-compartment model comparison of dual-energy x-ray absorptiometry and impedance-based devices.** J Strength Cond Res. 2020; 34 (6):1676-1689.	Full text n=49 Pubmed eligible - excluded athletes
Tanaka S, Ando K, Kobayashi K, Seki T, Hamada T, *et al.* R**eduction in body cell mass as a predictor of osteoporosis: A cross-sectional study.** Mod Rheumatol. 2020; 30 (2):391-396.	Full text n=49 scopus eligible but excluded - not 4C v BIA
Tinsley GM and Moore ML. B**ody fat gain and loss differentially influence validity of dual-energy x-ray absorptiometry and multifrequency bioelectrical impedance analysis during simultaneous fat-free mass accretion.** Nutr Res. 2020; 75 44-55.	Full text n=49 Pubmed eligible – excluded, body volume estimated by DXA

Tinsley GM. F**ive-component model validation of reference, laboratory and field methods of body composition assessment.** Br J Nutr. 2021; 125 (11):1246-1259.	Full text n=49 Pubmed eligible - excluded 5C model
Matias CN, Campa F, Santos DA, Lukaski H, Sardinha LB, *et al.* F**at-free Mass Bioelectrical Impedance Analysis Predictive Equation for Athletes using a 4-Compartment Model.** Int J Sports Med. 2021; 42 (1):27-32.	Full text n=49 Pubmed eligible but excluded, sport
Brewer GJ, Blue MNM, Hirsch KR, Saylor HE, Gould LM, *et al.* V**alidation of InBody 770 bioelectrical impedance analysis compared to a four-compartment model criterion in young adults.** Clin Physiol Funct Imaging. 2021; 41 (4):317-325.	Full text n=49 Pubmed eligible - included
Blue MNM, Hirsch KR, Brewer GJ, Cabre HE, Gould LM, *et al.* T**he validation of contemporary body composition methods in various races and ethnicities.** Br J Nutr. 2022; 128 (12):2387-2397.	Full text n=49 Pubmed eligible - included
Tinsley GM, Harty PS, Stratton MT, Smith RW, Rodriguez C, *et al.* T**racking changes in body composition: comparison of methods and influence of pre-assessment standardisation.** Br J Nutr. 2022; 127 (11):1656-1674.	Full text n=49 Pubmed eligible – excluded, no baseline data comparison
Schlösser L, Delgado FSG, Da Silva LV, Copetti CLK, Di Pietro PF, *et al.* V**alidity of body fat percentage through different methods of body composition assessment in elite soccer referees.** Rev Bras Cineantropometria Desempenho Hum. 2022; 24 e84121.	Full text n=49 Scopus eligible but excluded, BIA used in both TBW for 4C model as well as comparator
Foote DM, Berkelhammer M, Marone J and Horswill CA. C**ombining anthropometry and bioelectrical impedance to predict body fat in female athletes.** J Athl Train. 2022; 57 (4):393-401.	Full text n=49 Pubmed eligible - excluded, sport
Brandner CF, Tinsley GM and Graybeal AJ. S**martwatch-based bioimpedance analysis for body composition estimation: precision and agreement with a 4-compartment model.** Appl Physiol Nutr Metab. 2023; 48 (2):172-182.	Full text n=49 Pubmed eligible - included
Siedler MR, Rodriguez C, Stratton MT, Harty PS, Keith DS, *et al.* A**ssessing the reliability and cross-sectional and longitudinal validity of 15 bioelectrical impedance analysis devices.** Br J Nutr. 2023; 130 (5):827-840.	Full text n=49 Pubmed eligible – included, baseline only
Blue MNM, Tinsley GM, Hirsch KR, Ryan ED, Ng BK, *et al.* **Validity of total body water measured by multi-frequency bioelectrical impedance devices in a multi-ethnic sample.** Clin Nutr ESPEN. 2023; 54 187-193.	Full text n=49 Scopus eligible but excluded - TBW only not 4C v BIA
González-Arellanes R, Urquidez-Romero R, Rodríguez-Tadeo A, Esparza-Romero J, Méndez-Estrada RO, *et al.* P**redictive equations for fat mass in older Hispanic adults with excess adiposity using the 4-compartment model as a reference method.** Eur J Clin Nutr. 2023; 77 (5):515-524.	Full text n=49 Pubmed eligible but excluded - not 4C v BIA, predictive equations
Nickerson BS, Tinsley GM, Park KS, Nassar B, Czerwinski SA. **Evaluation of a Rapid Four-Compartment Model and Stand-Alone Methods in Hispanic Adults.** J Nutr. 2023;153(8):2154-2162.	Full text n=49 Pubmed eligible – included,

Supplemental Table S3a. Quality of assessment of included studies by Appraisal tool for Cross-Sectional Studies, Questions 1 to 11 (AXIS) [2]

		Methods									
	QR	QSD	QSD	QR	QSD	B	B	QSD	B	QR	QR
	1	2	3	4	5	6	7	8	9	10	11
**Fuller 1992**	Y	Y	N	Y	Y	Y	-	Y	Y	Y	Y
**Jebb 2000**	Y	Y	N	Y	Y	Y	-	Y	Y	Y	Y
**Chouinard 2007**	Y	Y	N	Y	Y	Y	-	Y	Y	Y	Y
**Korth 2007**	Y	Y	N	Y	Y	Y	-	Y	Y	Y	Y
**Gibson 2008**	Y	Y	N	Y	Y	Y	-	Y	Y	Y	Y
**Bosy-Westphal 2013**	Y	Y	N	Y	Y	Y	-	Y	Y	Y	Y
**Nickerson 2017**	Y	Y	N	Y	Y	Y	-	Y	Y	Y	Y
**Brewer 2021**	Y	Y	N	Y	Y	Y	-	Y	Y	Y	Y
**Blue 2022**	Y	Y	Y	Y	Y	Y	-	Y	Y	Y	Y
**Brandner 2023**	Y	Y	N	Y	Y	Y	-	Y	Y	Y	Y
**Siedler 2023**	Y	Y	Y	Y	Y	Y	-	Y	Y	Y	Y
**Nickerson 2023**	Y	Y	N	Y	Y	Y	-	Y	Y	Y	Y

Supplemental Table S3b Quality of assessment of included studies by Appraisal tool for Cross-Sectional Studies Questions 12 to 20 (AXIS) [2]

	Results					Discussion		Other	
	QR	B	B	B	QR	QSD	QR	QSD	QSD
	12	13	14	15	16	17	18	19	20
**Fuller 1992**	Y	-	-	Y	Y	Y	Y	N	Y
**Jebb 2000**	Y	-	-	Y	Y	Y	N	Y	Y
**Chouinard 2007**	Y	-	-	Y	Y	Y	Y	N	Y
**Korth 2007**	Y	-	-	Y	Y	Y	N	N	Y
**Gibson 2008**	Y	-	-	Y	Y	Y	Y	N	Y
**Bosy-Westphal 2013**	Y	-	-	Y	Y	Y	Y	Y	Y
**Nickerson 2017**	Y	-	-	Y	Y	Y	Y	N	Y
**Brewer 2021**	Y	-	-	Y	Y	Y	Y	N	Y
**Blue 2022**	Y	-	-	Y	Y	Y	Y	N	Y
**Brandner 2023**	Y	-	-	Y	Y	Y	N	N	Y
**Siedler 2023**	Y	-	-	Y	Y	Y	Y	N	Y
**Nickerson 2023**	Y			Y	Y	Y	Y	N	Y

QR = Quality of reporting, **QSD** = Quality of study design, **B** = possible bias

Supplemental Table S4. Stated pre-measurement study protocols

	**Pre-measurement protocol**
**Fuller 1992**	No mention of any pre-measurement protocols
**Jebb 2000**	"Subjects had not eaten for at least 2 h before the measurement and each emptied their bladder immediately before the start of the measurements."
**Chouinard 2007**	"All testing occurred in the morning within a 4-hour time period, at each of the 0- and 6-month visits. Subjects arrived after a 12-hour fast and were provided with a small breakfast bar"
**Korth 2007**	"Subjects arrived after an overnight fast at 0730 hours at the Institute for Human Nutrition and Food Science of the Christian-Albrechts-University, Kiel."
**Gibson 2008**	"On arrival at the Human Performance Laboratory (HPL) of Barry University, participants completed a brief health history questionnaire. Participants then voided, changed into dry swimwear (or athletic shorts and tee-shirt), and removed any remaining jewelry."
**Bosy-Westphal 2013**	"The subjects were asked to report to the study centres between 0700 and 0730 hours and 10 h after the last food and liquid intake."
**Nickerson 2017**	"Data collection for each subject occurred during the morning and afternoon hours (i.e., 9:00 AM–3:00 PM)."<br>"Subjects were asked to avoid eating or drinking, except water, 3 hours before participating in the study. In addition, subjects were asked to avoid exercise 12 hours before testing. Although standard protocol for BIA testing suggests subjects fast 8–12 hours before testing, Androutsos et al. (2) recently reported that eating and drinking had minimal practical implications on the assessment of body composition 2 hours after consumption. Thus, the current study chose to have subjects fast 3 hours before testing, which is more practical in a field setting application were BIA is often used."
**Brewer 2021**	"Subjects were required to arrive to the laboratory a minimum of eight hours fasted from food, caloric beverages, caffeine, alcohol and tobacco, but remain euhydrated. Additionally, subjects were required to abstain from strenuous exercise a minimum of twelve hours prior to testing. Inclusion criteria and adherence to pre-assessment guidelines were confirmed at the beginning of the visit."
**Blue 2022**	"For this cross-sectional, observational study, participants reported to the laboratory for a single testing session after a 12 h fast including abstention from caffeine, alcohol and tobacco. Participants refrained from strenuous exercise for a minimum of 24 h prior to testing. Following enrolment, participants provided a urine sample to assess hydration by urine specific gravity (for inclusion, urine specific gravity had to be between 1·002 and 1·029 measured by a refractometer)."
**Brandner 2023**	"Participants reported to the laboratory for testing after abstaining from food, beverages including water, supplements, medication, and exercise for ≥8 h. Participants were then instructed to void their bladder and their urine was compared with an 8-point color chart to verify hydration (urine color ≤ 6) (Graybeal et al. 2020)."
**Siedler 2023**	"All participants were instructed to abstain from exercise and vigorous physical activity for 24 h and to abstain from all food, fluid, caffeine, alcohol, nicotine or other substances for 8 h prior to each scheduled visit. To support adequate hydration during the visit, participants were also instructed to ingest 1 litre of water between their last meal and the beginning of the 8-h abstention from fluid. To further standardise measurements, participants wore skin-tight clothing (e.g. compression shorts, sports bra for females) for the duration of each visit. All visits were scheduled to commence between the hours of 6.00 and 14.00."<br>"At each visit, participants' adherence to the pre-testing guidelines was confirmed. Participants were then instructed to void their bladder. At this time, a urine sample was collected for the assessment of urine specific gravity using a digital refractometer (PA201X-093, Misco)."
**Nickerson 2023**	"Participants were asked to complete an overnight fasting protocol, which consisted of not eating or drinking 8 h prior to participation and to also avoid exercise 24 h before testing."

Supplemental Table S5. Abbreviated and modified Abu-Arafeh Bland-Altman Checklist on included studies [3]

Questions		**Fuller 1992**	**Jebb 2000**	**Chouinard 2007**	**Korth 2007**	**Gibson 2008**	**Bosy-Westphal 2013**	**Nickerson 2017**	**Brewer 2021**	**Blue 2022**	**Brandner 2023**	**Siedler 2023**	**Nickerson 2023**
**1**	A priori, establish the acceptable limit of agreement	no	no	no	no	no	no	no	no	no	no	no	no
**4**	Plot the data and inspect for absence of trend and constant variance	no	no	no	no	yes	yes	yes	yes	yes	yes	yes	yes
**6**	Plot[a] and report[b] numerically the mean of the differences (Bias)	b	a	a b	b	a b*	a b	a b	a b	a b	a b	a b	a b
**8**	Calculate and indicate the limits of agreement (LOA) of the differences	yes	yes	yes	yes	yes	yes	yes	yes	yes	yes	yes	yes
**10**	Ensure that the range of the mean values is sufficient	n/a	n/a	n/a	n/a	n/a	n/a	n/a	n/a	n/a	n/a	n/a	n/a
**12**	Software or computing processes used	yes	yes	yes	yes	yes	yes	yes	yes	yes	yes	yes	yes
14	*Correct representation of X-axis**	yes	yes	yes	yes	yes	yes	yes	yes	yes	yes	yes	yes
15	*Formal sample size calculations for BA*	no	no	no	no	no	no	no	no	no	no	no	no

- Plots of Gibson appear to use a “mean difference of zero” rather than plotting actual bias

**Additional questions

Supplemental Table S6. Summary of methodological heterogeneity across included studies

Domain	Variants observed across included studies
Four-compartment (4C) model equation	Fuller et al. (1992); Selinger (1977); Wang et al. (2002, 2005)*
Body volume (BV) method	Hydrodensitometry (underwater weighing); air-displacement plethysmography (BodPod); DXA-derived body volume (“rapid” 4C models)
Residual lung / thoracic gas volume handling	Helium dilution; oxygen dilution; manufacturer-derived thoracic gas volume (BodPod; software version–dependent)
Total body water (TBW) method	Isotope dilution (D_2O or tritium; saliva, blood or urine); bioimpedance spectroscopy (BIS; SFB7, Xitron Hydra)
Extracellular water (ECW) handling	Explicit ECW measurement (e.g. NaBr dilution) vs not measured or not reported
Bone mineral content (BMC) method	DXA only, but with different manufacturers and models (GE Lunar, Hologic, Norland) and different software versions
BIA measurement posture	Supine; standing (vertical)
BIA electrode configuration	Hand-to-foot; foot-to-foot; octopolar/segmental (hand and foot contact); handrail-based systems
BIA frequency profile	Single-frequency (typically 50 kHz); multi-frequency (ranges from 1–1000 kHz)
BIA prediction equations	Proprietary manufacturer equations; published regression equations applied post-hoc (multiple equations tested within the same study)
Participant body composition range	Lean/normal-weight cohorts; mixed BMI cohorts including overweight/obese participants
Sex and ethnicity handling	Male-only, female-only, or mixed-sex samples; predominantly White cohorts vs explicitly multi-ethnic samples
Agreement analysis reporting	Bland–Altman bias and limits of agreement reported in all studies, but with inconsistent availability of plots and inconsistent assessment of proportional bias
Multiplicity of comparisons	Several studies contributed multiple comparisons via testing multiple BIA devices, equations, or subgroup analyses (sex or ethnicity), increasing within-study heterogeneity

*Wang 2002 and Wang 2005 are the same formula

References

1. Clark JM, Sanders S, Carter M, Honeyman D, Cleo G, Auld Y, et al. Improving the translation of search strategies using the Polyglot Search Translator: a randomized controlled trial. J Med Libr Assoc. 2020;108(2):195-207. doi: 10.5195/jmla.2020.834.
2. Downes MJ, Brennan ML, Williams HC, Dean RS. Development of a critical appraisal tool to assess the quality of cross-sectional studies (AXIS). BMJ Open. 2016;6(12):e011458. doi: 10.1136/bmjopen-2016-011458.
3. Abu-Arafeh A, Jordan H, Drummond G. Reporting of method comparison studies: a review of advice, an assessment of current practice, and specific suggestions for future reports. Br J Anaesth. 2016;117(5):569-75. doi: 10.1093/bja/aew320.
